# Induced pluripotent stem cell-based models: Are we ready for that heart in a dish?

**DOI:** 10.3389/fcell.2023.1129263

**Published:** 2023-01-19

**Authors:** Irene Bissoli, Stefania D’Adamo, Carla Pignatti, Giulio Agnetti, Flavio Flamigni, Silvia Cetrullo

**Affiliations:** ^1^ Department of Biomedical and Neuromotor Sciences, Alma Mater Studiorum, University of Bologna, Bologna, Italy; ^2^ Istituto Nazionale per le Ricerche Cardiovascolari, Bologna, Italy; ^3^ Center for Research on Cardiac Intermediate Filaments, Johns Hopkins University, Baltimore, MD, United States

**Keywords:** human induced pluripotent stem cells (hiPSCs), hiPSC-derived cardiomyocytes, cardiovascular disease modelling, cardiomyocyte maturation, engineered heart tissues (EHTs), heart-on-a-chip, cardiac organoids

## 1 Introduction

A major limitation in studies aimed at investigating the molecular bases and pathogenetic mechanisms in cardiac disease is the limited availability of cellular models capable of reproducing the characteristics of the disease in humans. While several *in vivo* models of cardiac disease spanning small and large animal models are available to date (Patten and Hall-Porter, 2009; Tsang et al., 2016), the bulk of *in cellulo* studies is based on primary cultures and cell lines (Savoji et al., 2019). All these models are certainly useful, but each of them has limitations which likely contributed to the limited translatability of *in cellulo* models. Indeed, two limitations with primary cultures from adult animals are their short viability once plated, and operator-dependent quality. On the other hand, the main shortcomings with cell lines or primary cultures obtained from neonatal animals (especially rat and mouse) are likely their lack of ultrastructure and immature metabolism.

**FIGURE 1:**
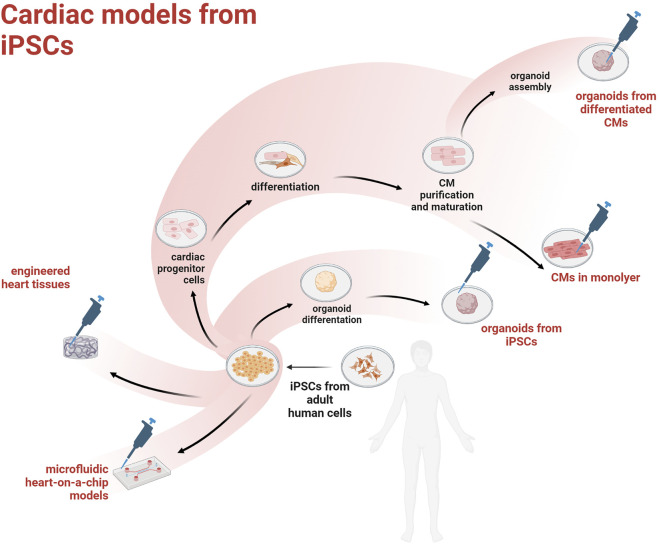
Overview of the main hiPSC-derived cardiac models.

The discovery of human induced pluripotent stem cells (hiPSCs) came with the promise to model many different human diseases in a dish and study the underlying cell pathobiology, or even establish *in vitro* assays for drug discovery/toxicity. Given that cardiovascular disease (CVD) is the largest killer worldwide, the optimization of protocols to obtain hiPSC-derived cardiac myocytes (hiPSC-CMs) has received a lot of attention and funding. However, the heart is a complex organ comprising an ever growing number of cell types and a unique spatial architecture needed to sustain the efficient propagation of depolarization and synchronous contraction. Overall, the translational value of mouse models has proven low (Nerbonne, 2004) and recent improvements in tissue engineering could help overcoming current limitations associated with the use of hiPSCs in cardiovascular research. Current main strategies to obtain cardiac models from hiPSCs are outlined in Figure 1.

In this viewpoint we address current limitations with these models as well as future promising avenues to create “hearts in a dish.”

## 2 Human induced pluripotent stem cells to model disease *in cellulo*

Fifteen years ago, among twenty-four candidate genes, Takahashi and Yamanaka *et al.*, identified a combination of key transcription factors sufficient to reprogram human adult dermal fibroblasts into hiPSCs (Takahashi et al., 2007). These four genes, also dubbed Yamanaka’s factors, are: 1) octamer-binding transcription factor 4 (OCT4); 2) Sry box-containing factor 2 (SOX2); 3) Kruppel Like Factor 4 (KLF4) and 4) c-MYC. Also an alternative panel of genes was identified as essential to induce pluripotency, namely Thomson’s factors, including Nanog and LIN28 with OCT4 and SOX2 (Yu et al., 2007). Moreover, in a subsequent study the four Yamanaka’s factors were reduced to three with the exclusion of c-MYC (Nakagawa et al., 2007). After being subjected to these optimized de-differentiation protocols, similar to embryonic stem cells, adult cells regain most of their pluripotency and can be thus differentiated into multiple lineages including cardiomyocytes. Nowadays different cell types can be de-differentiated to obtain hiPSCs, including dermal fibroblasts, as in the first experience of reprogramming (Takahashi et al., 2007), and peripheral blood cells like T cells (Seki et al., 2010). Integrative (retrovirus and lentivirus) and nonintegrative (Sendai virus, episomal vectors and RNA transfection) vectors can be employed to introduce transgenes needed to induce pluripotency, with various levels of efficiency and biosafety requirements, but with essentially similar results (Schlaeger et al., 2014).

hiPSCs are particularly relevant to human cardiac pathophysiology in that they are *de facto* human and can be obtained from patients carrying disease-driving mutations, thus providing cell lines that can be used to investigate the alterations caused by a specific genetic background. This feature is clearly important for personalized medicine. In addition, recent advances in gene editing techniques, including clustered regularly interspaced short palindromic repeats (CRISPR)-associated (Cas) systems, have allowed scientist to introduce specific mutations in “wild-type” hiPSCs in order to model inheritable cardiomyopathies or create isogenic controls from hiPSCs obtained from patients carrying specific mutations (Matsa et al., 2016). Isogenic controls allow to narrow down differences to the specific mutation, thus reducing confounding factors such as heterogenous genetic background that could affect other cellular models (e.g., cell lines). These strategies so far were deployed to model various CVD-causing genetic mutations, including but not limited to LEOPARD (Carvajal-Vergara et al., 2010) and long-QT syndromes (Moretti et al., 2010), dilated cardiomyopathy due to mutation in the *PPCS* gene (Iuso et al., 2022), and a newly reported mutation in the *DES* gene, causing restrictive cardiomyopathy (Brodehl et al., 2019).

## 3 Current limitations and promising avenues with the use of hiPSCs in cardiac models

The most widely used protocols to obtain CMs in a monolayer from hiPSCs are based on the inhibition of glycogen synthase kinase-3β and Wnt (Lian et al., 2012; Burridge et al., 2014). Indeed, recapitulating what takes place during cardiogenesis, the inhibition of this signal pathway is usually required to exit pluripotency and to generate mesodermal progenitors, which are able to subsequently commit to cardiac cell lineages including ventricular and atrial CMs, pacemaker and conduction cells, endothelial cells, fibroblasts, pericytes, smooth cells and resident immune cells.

Sometimes it is preferable to use a reductionist approach and focus on specific cell populations in order to pinpoint a pathological mechanism in greater detail, rather than reproduce the complete heterogeneity of cardiac tissue. This allows to focus on specific aspects if a pathological phenotype is known as associated with alterations of a particular cell type. For example, many heart diseases such as hypertrophic cardiomyopathy (Lan et al., 2013) or metabolic disorders like diabetic cardiomyopathy (Drawnel et al., 2014) are usually modelled by ventricular CM cultures. In all these cases, it should be taken in consideration that not a complete purification, but an enrichment of a cellular type is currently achievable. One of the most used protocols to obtain a very high purification (almost 99%) of hiPSC-CMs is based on their ability to metabolize lactate in a glucose-deprived medium (Tohyama et al., 2013), however, this property does not necessarily select among subtypes of CMs (e.g. atrial, ventricular and nodal).

Although current protocols allow to obtain hiPSC-CMs with high purity, several features observed in adult CMs are not easily reproduced in differentiated cells. For start, adult CMs are typically polynucleated also due to their larger size/volume and have typically elongated (rather than spheroidal) shape. In addition, adult CMs contain numerous mitochondria layered between highly organized sarcomere which align at the level of the z-discs. Currently, the dense functional ultrastructure of adult CMs cannot simply be recapitulated in hiPSC-CMs posing major limitations to biophysical studies, including the assessment of excitation-contraction coupling with standard techniques (Sayed et al., 2016; Besser et al., 2018). Lastly, the use of energetic substrates by hiPSC-CMs is profoundly different from adult CMs, which largely prefer fatty acids instead of glucose as their main fuel for ATP production (Lopaschuk et al., 2021; Vučković et al., 2022). This feature has obvious implication not only for CVD caused by metabolic imbalance (both genetic and environmental), but likely CVD at large.

Overall, it is fair to assume that at the current stage hiPSC-CMs are more similar to embryonic CMs and thus less differentiated than established primary cultures from neonatal rat ventricular myocytes (Robertson et al., 2013; Sayed et al., 2016; Heffler et al., 2020). Exposure to both chemical and physical stimuli (Mihic et al., 2014; Yang et al., 2014; Zhang et al., 2017; Karbassi et al., 2020) or co-culture with non-cardiomyocytes (Kim et al., 2010) are examples of strategies that were recently implemented to improve this glaring issue with hiPSC-CMs. Prolonged time in culture (up to 120 days) also seems to improve morphological and electrophysiological maturity of hiPSC-CMs (Lundy et al., 2013). Lastly, newly optimized maturation media have been formulated to promote the use of substrates typical of mature CMs (Feyen et al., 2020). As mentioned, the metabolic switch from glucose to fatty acid utilization in hiPSCs is paramount to modelling adult disease.

Notably, 3D cultures such as engineered heart tissues (EHTs) and organoids, are rapidly gaining traction for their capacity of improving a more adult-like phenotype in hiPSC-CMs (Tulloch et al., 2011; Nunes et al., 2013; Tiburcy et al., 2017). EHTs can be generated by layering different types of cardiac cells on various biomaterials (including de-cellularized cardiac matrices) to provide spatial orientation and recapitulate a more physiological paracrine environment. Over the years different geometries for EHTs including but not limited to strips, patches, rings, films, and spheroids have been tested [for an overview (Cho et al., 2022)]. In some of these models hiPSCs are addressed to differentiate before the tissue assembly, other studies used undifferentiated cells postponing the process in the 3D structure. Topographical cues on scaffolds, like anisotropic patterning, microgrooves and microridges can influence cell shape and alignment so adding a strong stimulus from the microenvironment for cardiomyocyte maturation (Carson et al., 2016).

One current limitation with EHTs is the lack of interlaboratory standardization. Luckily, the private sector is addressing this gap by producing standardized matrices (e.g., decellularized porcine cardiac matrix), as well as devices which are able to measure a number of functional parameters including alterations in Frank-Starling curve, length-dependent activation and others (Schwan et al., 2016; Shen et al., 2021). These “plug-and-play” EHTs allow to populate the available matrices with hiPSC-CMs of choice and therefore compare genotypes and drug treatments under standardized settings.

An emerging model to recapitulate and monitor organ function is represented by microfluidic heart-on-a-chip platforms, which are rapidly becoming available with diverse configurations and capabilities. Regarding that, we are likely just witnessing the dawn of a new era in EHT technology. As summarized in a recent review (Paloschi et al., 2021), organs-on-a-chip converge cell biology, nanomaterials, and fabrication technologies to monitor the mechanical and electrical cues of the cells *in vitro* and mimic the spatiotemporal microenvironment of the tissue. Typically, these systems integrate 2D cell cultures on permeable membranes or cells cultured in 3D hydrogel scaffolds. It should be noted that the material most used for the microfluidic channels is poly(dimethylsiloxane) (PDMS). Despite many advantages that make it a convenient material (such as optical transparency, ease of manufacture and gas permeability), PDMS can adsorb hydrophobic compounds to some extent (van Meer et al., 2017). This must be considered as a possible confounding factor in drug screening studies.

These microdevices allow to set up a dynamic tissue culture to control specific microphysiological conditions, also combining electrical, mechanical, and topographical stimuli and eventually integrating biosensors to obtain a real-time and continuous monitoring of dynamic tissue behaviors (Criscione et al., 2023). Ideally, different organs-on-a-chip in series will be able to recapitulate the systemic effects of drugs across multiple organs (Skardal et al., 2020; Picollet-D’hahan et al., 2021). Perhaps, these multiorgan-on-a-chip could improve the accuracy of individualized drug response and side effects in preclinical settings.

Organoids by definition are self-assembling structures that recapitulate the events taking place during the first stages of life. The feature of hiPSCs to form embryoid body-like structures is exploited by these models. Several combinations of inhibitors and growth factors direct this ability towards the cardiac differentiation, reproducing a complex structure with multiple cell lineages like in *in vivo* cardiogenesis. The advantage of organoids over conventional EHTs is the possibility of benefitting from the interaction among the different cell types in a more “physiological” microenvironment. In some models the presence of an extra-cellular matrix support [like Matrigel (Drakhlis et al., 2021), laminin or vitronectin (Hofbauer et al., 2021)] is required to promote the correct organization, while other systems can organize autonomously (Lewis-Israeli et al., 2021). A recent study has compared the ability of cardiac mesoderm cells and cardiomyocytes, derived respectively from 4 to 11 day of differentiation of hiPSCs, to generate cardiac organoids. This study found that the former protocol resulted in the improvement of structure, metabolism, function and overall maturation (Song et al., 2021). In both cases, it is important to note that cardiac organoids arise from a single cell population that self-organizes, unlike cardiac spheroids which are assembled mixing different cell populations (cardiomyocytes, endothelial cells and fibroblasts) at defined ratios (Polonchuk et al., 2017). More recently a new strategy to improve maturity and complexity in cardiac organoids has been proposed as able to reproduce anterior-posterior heart tube patterning by endogenous retinoic acid signaling and self-organization (Volmert et al., 2022).

A very recent paper showed the generation of cardioids with compartment-specific features recapitulating the main structures such as right and left ventricles, atria, outflow tract, and atrioventricular canal (Schmidt et al., 2022). This human cardioid platform allows the modelling of early specification, morphogenesis, and signal contraction propagation of the human embryonic heart.

There have been reports of mini-heart constituted by either one (Hofbauer et al., 2021) or two chambers (Lewis-Israeli et al., 2021) with structural properties reminiscing of the organ but limited pump function and therefore, output flow. In order to characterize this kind of 3D models, as previously done with tumoral spheroids, a customized spectral-domain optical coherence tomography system can be employed to improve the ability to monitor the *in vitro* system during the development phases in a non-destructive manner (Ming et al., 2022). It should be underscored that the size of complex 3D structures is currently self-limited by the formation of an adequate vascular bed, which would replace passive oxygen and nutrients diffusion as the structure becomes bigger.

In all, while stem cells currently constitute a powerful tool in the hands of developmental biologists, the use of hiPSC-CMs to model CVD and discover new pathological mechanisms, and new pharmaceutical targets, is still in its embryonic phase (no pun intended). We are confident that further advancement in tissue engineering and new knowledge on endogenous and exogenous contributors to the maturation of cardiac structure and metabolism will soon open new avenues to model CVD and test new therapies in a dish.
